# Prevalence and Sociodemographic Factors Associated with Overweight and Obesity among Adults in Poland: A 2019/2020 Nationwide Cross-Sectional Survey

**DOI:** 10.3390/ijerph19031502

**Published:** 2022-01-28

**Authors:** Katarzyna Stoś, Ewa Rychlik, Agnieszka Woźniak, Maciej Ołtarzewski, Mateusz Jankowski, Mariusz Gujski, Grzegorz Juszczyk

**Affiliations:** 1 Department of Nutrition and Nutritional Value of Food, National Institute of Public Health NIH—National Research Institute, 00-791 Warsaw, Poland; awozniak@pzh.gov.pl (A.W.); moltarzewski@pzh.gov.pl (M.O.); 2 Centre of Postgraduate Medical Education, School of Public Health, 01-826 Warsaw, Poland; mjankowski@cmkp.edu.pl; 3 Department of Public Health, Medical University of Warsaw, 02-097 Warsaw, Poland; mariusz.gujski@wum.edu.pl (M.G.); grzegorz.juszczyk@wum.edu.pl (G.J.)

**Keywords:** overweight, obesity, weight status, epidemiology, adults, Poland

## Abstract

Detailed characteristics of the weight status of the population is necessary for the effective prevention of health disorders, e.g., through personalized nutrition education. This study aimed to characterize weight status and identify sociodemographic factors associated with overweight/obesity in a representative sample of adult inhabitants of Poland. This cross-sectional study was carried out from July 2019 to February 2020 on a representative nationwide sample of individuals aged 18+ in Poland. The study consisted of two parts: questionnaire survey and anthropometric measurements. The body mass index was calculated. Data on 1831 adults (50.3% females; mean age 51.7 ± 19.9 years) were included in this analysis. The prevalence of overweight was 42.2% (52.4% among males and 32.0% among females). Of the 1831 participants, 16.4% were obese (16.5% of males and 16.2% of females). Out of 11 factors analyzed in this study, only 5 were significantly associated with overweight/obesity. Males, older participants, occupationally active individuals, those living in rural areas and individuals with at least one chronic disease had greater odds of overweight/obesity. This study demonstrated a high prevalence of overweight and obesity among adults in Poland. This is the most up-to-date representative study on nutritional status carried out before the COVID-19 pandemic.

## 1. Introduction

According to the World Health Organization (WHO), overweight and obesity are global public health problems that have become global epidemics [1]. Overweight and obesity result from excessive accumulation of fat in the body because of an energy imbalance between calories consumed and calories expended [2]. Dietary habits, physical activity level, genetics, environmental factors, and socioeconomic status have contributed to overweight and obesity [2,3,4]. Moreover, changes in dietary and physical activity patterns are often the result of social, economic, and nutritional transitions related to the country’s development, urbanization, and industrialization [5,6]. In 1989, Poland embraced a transition to democracy, and in 2004 joined the European Union (EU), which had a positive impact on the standard of living and access to goods and services [7]. According to the WOBASZ survey, some lifestyle factors changed in Poland in the years 2003–2014. These were positive changes (a greater proportion of non-smokers and those following a good-quality diet) and negative changes (a lower proportion of people with an adequate physical activity level and low consumption of saturated fat) [8].

Overweight and obesity increase the risk of numerous chronic diseases such as, above all, cardiovascular diseases, type 2 diabetes, sleep apnea, some musculoskeletal conditions, and certain types of cancer [9,10]. Overweight and obesity are serious health hazards, so nutritional status both at the individual as well as the populational level should be regularly monitored. 

The body mass index (BMI) is a simple index of weight-for-height that is commonly used in population-based studies to assess weight status and classify overweight and obesity in adults [11,12]. The WHO estimates that more than 1.25 billion adults are overweight, defined by WHO as a body-mass index (BMI) of ≥25.0 kg/m^2^, and of these over 650 million are obese (BMI ≥ 30 kg/m^2^) [13]. 

Poland is a high-income country in Central and Eastern Europe that has undergone a significant transition over the past thirty years [14]. After joining the European Union (EU) in 2004, numerous social and economic changes occurred that have a direct impact on dietary behaviors (e.g., through European Single Market as well as General Food Law Regulations) [15]. In accordance with the Statistics Poland data, the gross domestic product (GDP) was 70% higher in 2020 in comparison to 2005—the first year after the accession of Poland to the EU [16]. Between 1997 and 2017, the prevalence of overweight had increased from 38% to 47.3% among males and from 30% to 32.2% among females [17]. At the same time, the prevalence of obesity had increased from 16% to 17.9% among males but decreased from 19% to 16.1% among females [17]. Factors that contributed to changes in the prevalence of overweight/obesity in Poland have not been studied sufficiently. 

Most of the epidemiological studies on the prevalence of overweight and obesity among adults in Poland are limited to selected towns or regions (voivodships) [18]. Between 1983 and 2005, there were only 9 countrywide studies on overweight and obesity occurrence in Poland. Moreover, only a part of these studies was representative, carried out based on anthropometric measurements [18]. Recently published studies on weight status in Polish adults are focused on dietary habits or lifestyle factors coexisting with increased body mass index [18,19]. There is a significant gap in nationwide representative studies on nutritional statuses in different socio-economic groups in Poland. Data from other countries indicate that sociodemographic factors may influence the risk of overweight and obesity [20,21]. It is particularly important to investigate the relationship between these factors and overweight and obesity in Poland due to significant changes in inter alia, age structure, level of education, and economic situation in recent years [22,23,24].

The aim of this study was to characterize weight status using anthropometric variables, as well as to identify sociodemographic factors associated with overweight/obesity in a representative sample of adult inhabitants of Poland.

## 2. Materials and Methods

### 2.1. Study Design

This cross-sectional study was carried out from July 2019 to February 2020 on a representative nationwide sample of 1831 individuals aged 18+ in Poland. The study consisted of two parts: questionnaire survey and anthropometric measurements. The computer-assisted personal interviewing (CAPI) technique was used [25]. Anthropometric measurements were made at the respondent’s home by qualified personnel under appropriate conditions and using certified devices.

All the interviews were carried out by a specialized survey company (subcontractor) on behalf of the Institute of Food and Nutrition, which has been included since the 1 February 2020 into the National Institute of Public Health NIH—National Research Institute (NIPH NIH—NRI), which provides the context of this research. 

This study was carried out as a part of Nationwide Dietary Survey in Poland which was conducted according to the European Food Safety Authority (EFSA) guidance on the EU Menu methodology [26,27]. The subcontractor met the quality requirements of EFSA.

### 2.2. Population

The respondents were adult inhabitants of Poland aged 18–96 years, randomly selected from the PESEL register (the national register of inhabitants in Poland) by a subcontractor [28]. A stratified sampling method was applied. The stratification model includes gender (females and males), age (7 age cohorts), as well as the size of the place of residence, and the territorial distribution within voivodships (16 voivodships, nine size classes of localities). The first part of the sample selection procedure was the stratification of the Polish population due to the criterion of territorial location and size class of localities. The localities were divided into nine size categories. The selected localities were the units of the random selection of subjects in the next part of the study. The next part of the sample selection procedure was the random selection of individuals from the previously selected localities, considering the criterion of gender and age. Within each stratum, subjects were selected by a systematic sampling method. 

To ensure the representativeness of the study sample, the size of the sample selected by the stratified sampling method was almost tenfold as high as assumed. If the respondent refused to participate in the study, withdrew from the study or met the exclusion criteria, another person from the group was selected. The interviewers were obliged to repeatedly try to contact a randomly selected respondent until the interview was successfully completed or it was stated that it could not be completed definitively (hard refusal, prolonged absence of the respondent during the survey).

Data on 2432 respondents were collected (response rate: 57.2%). This study was carried out as a part of Nationwide Dietary Survey in Poland carried out both among children and adults, but data on adolescents aged 10–17 years were excluded from this analysis (*n* = 601). In total, 1831 adult inhabitants of Poland were included in this study. 

Participation in the study was voluntary. Written informed consent was obtained from each respondent. The study protocol was reviewed and approved by the Bioethics Committee at the Institute of Food and Nutrition in Warsaw, Poland (opinion dated 4 June 2018).

### 2.3. Study Questionnaire

#### 2.3.1. Sociodemographic Factors

Socio-demographic data were collected using a questionnaire developed for the purpose of this study. Questions addressed personal characteristics, including gender (male or female), age (years), marital status, place of residence, number of household members, having children, educational level, occupational status as well as financial situation. Participants were also asked about the presence of chronic diseases and self-reported health status. In the case of the age criterion, the following was applied: 18–29 years, 30–39 years, 40–49 years, 50–59 years, 60–69 years and 70+ years. Marital status was classified as single, married, divorced, or widowed. Place of residence was classified as rural or urban. Educational level was classified as primary education, vocational education, secondary education or higher education. The occupational activity was classified as active (currently employed) or passive (currently unemployed) occupational status. The financial situation was assessed according to the 5-item scale: very good, good, moderate, bad, very bad. The health status (concerned both physical and mental health) was assessed based on the questionnaire used by the Statistics Poland in the European Health Interview Survey (EHIS) [22]. The following question was used: “How is your health in general?” with five possible answers: very good, good, moderate, bad or very bad. There was no failure to respond.

#### 2.3.2. Physical Activity Assessment

Respondents’ physical activity level was assessed using the short version of International Physical Activity Questionnaire (IPAQ; Polish version) [29]. Respondents were categorized into low, moderate, and high physical activity level groups according to the scoring rules of the IPAQ scoring protocol [30].

### 2.4. Anthropometric Measurements 

Anthropometric measurements were collected by qualified research team member. The anthropometric measurements took place at respondent’s home. The height and weight of the respondents were measured using the bodymeter (Seca, Hamburg, Germany) ensuring the accuracy of 0.1 cm and electronic scales (Soehnle, Backnang, Germany) with the accuracy of 0.1 kg. Body mass index (BMI) was calculated as weight divided by height squared (kg/m^2^). The BMI categories were identified according to international standards [31,32]. Respondents were categorized into three BMI groups: normal-weight (≥18.5–24.9 kg/m^2^), overweight (≥25.0–29.9 kg/m^2^), and obese (≥30.0 kg/m^2^). In obese subjects, the prevalence of obesity of different classes was also analyzed: class I (BMI ≥ 30.0–34.9 kg/m^2^), class II (≥35.0–39.9 kg/m^2^), and class III (≥40.0 kg/m^2^). Data on underweight respondents (BMI < 18.5 kg/m^2^; *n* = 20) were only used to assess the prevalence of each BMI groups in the population and excluded from the further analysis because this study focuses on the factors associated with overweight and obesity. For the purposes of some analysis, categories overweight and obesity were combined into one category (BMI ≥ 25.0 kg/m^2^).

### 2.5. Statistical Analysis

The data were analyzed with SPSS version 27 software (IBM, Armonk, NY, USA). The distribution of categorical variables was shown by frequencies and proportions. Statistical testing to compare categorical variables was completed using the independent samples chi-square test. Due to limited number of papers on weight status in Polish population, sociodemographic characteristics of the participants were presented in two ways: overall characteristics with division into two categories (normal-weight; overweight/obese) and detailed characteristics by gender with division into three categories (normal-weight; overweight; obesity).

Unadjusted and adjusted odds ratio (OR) were estimated using univariate and multivariate logistic regression analysis, respectively. Unrestricted (full) multivariate logistic regression analysis examined the effect of sociodemographic factors on overweight/obesity after adjustment, while restricted multivariate logistic regression analysis selected only independent factor using step-by-step backward method.

The strength of association was measured by the odds ratio (OR) and 95% confidence intervals (CI). Statistical inference was based on the criterion *p* < 0.05.

## 3. Results

### 3.1. Characteristics of the Sample

Responses were received from 1831 adults (50.3% females; mean age 51.7 ± 19.9 years; 51.7 ± 19.8 among males and 51.7 ± 19.8 among females). The prevalence of overweight was 42.2% (52.4% among males and 32.0% among females). Of the 1831 participants, 16.4% were obese (16.5% of males and 16.2% of females). Class I obesity was the most common, it occurred in 14.0% (14.3% of males and 13.7% of females). There were fewer subjects with class II obesity (2.1%), and very few with class III obesity (0.3%). Among the participants, 1.1% (0.2% of males and 2.0% of females) were underweight (BMI < 18.5 kg/m^2^). Underweight respondents (2 males and 18 females) were excluded from the further analysis because this study focuses on the factors associated with overweight and obesity. Table 1 shows the characteristics of the study sample classified by BMI status. 

The percentage of overweight/obese individuals increased with age (*p* < 0.0001). The percentage of overweight/obese individuals differed by marital status, educational level, occupational status, financial situation, having children under 18 years of age, as well as health status (Table 1). There were no statistically significant differences in the prevalence of overweight/obesity depending on the physical activity level (*p* = 0.58). 

### 3.2. Sociodemographic Differences in the Prevalence of Overweight and Obesity among Males and Females

The prevalence of overweight and obesity among males and females by sociodemographic factors is presented in Table 2. More than half of males and almost a third of females were overweight. Both among males and females, the percentage of overweight/obese individuals increased with age (*p* < 0.001). Moreover, both among males and females, there were significant differences in the percentage of overweight or obese individuals by marital status, educational level, financial situation, living conditions, self-reported health status/presence of chronic diseases (Table 2). Among females, there were significant differences in the percentage of overweight or obese individuals by occupation status and having children under 18 years of age. These differences were not observed among males. Both among males and females, there were no significant differences in the prevalence of overweight and obesity depending on the physical activity level. In contrary to the full sample, occupational status and having children under 18 years of age were not statistically significant when splitting by gender.

In this study, regardless of the BMI status, males reported a higher prevalence of high physical activity level compared to females. However, among individuals with normal weight, there were no statistically significant differences in the physical activity level between males and females. Among overweight/obese individuals, females significantly more often declared low physical activity level compared to males (25.7% vs. 18.8%; *p* = 0.01). Details are presented in Table 3.

### 3.3. Factors Associated with Overweight/Obesity among Adults in Poland 

The results of the univariate and multivariate logistic regression analyses are presented in Table 4. In a restricted multivariate logistic regression model, five factors were associated with increased odds of overweight/obesity (see Table 4). 

The odds of overweight/obesity increased with age (OR: 1.04; 95% CI = 1.03–1.04; <0.01). Males had almost two and half times higher odds of overweight/obesity (OR = 2.44, 95% CI = 2.00–2.99; *p* < 0.001). Those who were occupationally active had higher odds of overweight/obesity (OR = 1.50; 95% CI = 1.17–1.93; <0.001) compared to occupationally passive individuals. Adult inhabitants of Poland who lived in rural areas had higher odds of overweight/obesity compared to those who lived in urban areas (OR = 1.32; 95% CI = 1.07–1.63; *p* = 0.008). Moreover, individuals with at least one chronic disease had significantly higher odds of overweight/obesity (OR = 1.51; 95% CI = 1.11–2.07; *p* = 0.009).

## 4. Discussion

To the best of the authors’ knowledge, this is the most up-to-date epidemiological study on the prevalence of overweight/obesity based on the anthropometric measurements and factors associated with overweight/obesity in a representative sample of adult inhabitants in Poland. Characteristics of the weight status carried out in this study showed, that more than half of adults in Poland are overweight or obese. Out of 11 factors analyzed in this study, only 5 were significantly associated with overweight/obesity. Males, older participants, occupationally active individuals, those living in rural areas and individuals with at least one chronic disease had greater odds of overweight/obesity.

According to the WHO’s global estimates, the prevalence of overweight and obesity among adults is similar among women and men (40% vs. 39%); however, the percentage of obese individuals is higher among women than among men (15% vs. 11%) [1]. In Poland, the prevalence of excess body weight is higher among men than among women [18,19]. A review of the countrywide representative research, including anthropometric measurements, showed that between 1997 and 2005, the incidence of overweight was estimated to be 38–41% among men and 28–30% among women, while the percentage of obese men and women was 16–21% and 19–22% [18]. In the years 2013–2014, the prevalence of overweight was 43.2% in men and 30.5% in women, while the prevalence of obesity was 24.4% and 25.0%, respectively [33]. A study on diet-related factors, physical activity, and weight status in Polish adults showed that, in 2016, the prevalence of overweight/obesity was almost 1.5 times higher among men (60.2%) than women (39.8%) [19]. This study showed that 68.9% of adult men and 48.2% of adult women in Poland had overweight or obesity. Due to significant differences in weight status between men and women, detailed sociodemographic characteristics by BMI for both genders were provided in this study. Both among men and women, there were significant differences in the percentage of overweight or obese individuals by age, marital status, educational level, financial situation, living conditions, self-reported health status/presence of chronic diseases.

This study showed that males had almost two and half times higher odds of being overweight/obese. This finding is in line with previous studies carried out among adults in Poland [19]. We can hypothesize that gender disparities in overweight/obesity may result not only from the different eating habits but also from differences in health and body image perception [34]. It seems that healthcare professionals should use other gender-specific arguments to persuade the patient to change their lifestyle and nutrition habits.

Moreover, in this study, the risk of overweight/obese individuals increased with age, which is in line with previous national and international studies [18,19]. Age is a factor that determines lifestyle, including eating behaviors, food choices and physical activity [35]. Overweight and obesity increase the risk of numerous non-communicable diseases. The disease burden also increases with age. Overweight/obesity in older adults may multiply their health problems that also affect health care use, disability, and workability [36].

Lifestyle factors have a significant impact on an individual’s health status [37]. Incorrect dietary habits and low physical activity, which depend on individual health choices, are the most important risk factors for overweight and obesity [1,2,3,4,5]. However, numerous studies underline the relationship between overweight/obesity and sociodemographic status [3,4,5,20,21,38,39]. Financial situation, educational level and occupational status are considered as the most important sociodemographic factors which contribute to overweight/obesity [38,39].

In our study, occupationally active individuals had higher odds of being overweight/obese. Access to food in the workplace and eating regularities can affect weight status. Stress at work can also affect eating habits. The relationship between the subjective assessment of occupational stress and eating habits was confirmed in a study conducted in Łódź, Poland [40]. More unhealthy eating patterns were observed in employees with higher levels of stress. However, recent findings showed that although total and leisure-time physical activities have beneficial effects on health and mortality, the occupational activity would have negative effects (“physical activity paradox”) [41,42]. Holtermann et al. showed that higher leisure-time physical activity is associated with reduced major adverse cardiovascular events and all-cause mortality risk, while higher occupational physical activity is associated with increased risks, independent of each other [41]. Findings from the systematic review with meta-analysis published by Coenen et al. indicate the need for further investigation on the mechanisms of the “physical activity paradox”, and suggests that physical activity guidelines may differentiate between occupational and leisure-time physical activity [42].

The mechanisms behind this should be explored further. If the observed associations are causal, then PA guidelines should differentiate between occupational and leisure time PA.

It is estimated that approximately 40% of Polish inhabitants live in rural areas [43]. Previous studies reported urban–rural differences in eating habits and physical activity [44,45]. There is a limited number of representative studies on the nutritional characteristics of Poles living in rural or urban areas. Jarosz et al. showed that among males, overweight and obesity were more common in urban areas, while among females they were more common in rural areas [18]. Most of the previously published studies are limited to school-aged children and adolescents [18,46]. However, the results of these studies are inconclusive. This study showed that adult inhabitants of Poland who lived in rural areas had higher odds of overweight/obesity compared to those who lived in urban areas. This observation may result from differences in lifestyle between individuals from urban and rural areas.

Patients with obesity are at major risk of developing a wide range of comorbid conditions, such as cardiovascular diseases, gastrointestinal disorders, type 2 diabetes, respiratory diseases, depression, and several types of cancer [47]. Otherwise, some diseases (e.g., hypothyroidism) and medications (e.g., antidepressants, antihyperglycemics, antihypertensives) are associated with significant weight gain [48]. In this study, individuals with at least one chronic disease had significantly higher odds of overweight/obesity. As obesity is a risk factor for many diseases [7,49], some subjects may first become obese and then develop other non-communicable diseases. We can also hypothesize, that people with worse health are less likely to be physically active which may contribute to weight gain. However, due to the study design (cross-sectional survey), this observation requires further investigation.

In contrary to previously published papers [19,50,51], this study revealed a lack of statistically significant association between odds of overweight/obesity and (1) educational level, (2) financial situation, (3) marital status, (4) having children under 18 years of age, (5) living alone or with other persons, and (6) physical activity level. Education is a fundamental social determinant of health, but in this study, there was no significant impact of higher education on the odds of overweight/obesity [52]. Household income is considered as a factor significantly associated with higher rates of eating vegetables, better eating habits, and using the information on nutrition labels [53]. However, in this study, the financial situation was not significantly associated with the risk of overweight/obesity. Moreover, the physical activity level was not significantly associated with overweight/obesity, which may result from the limited access to sports venues outside large cities and the lack of proper education on physical culture since primary school. Between 2004 and 2019, the percentage of individuals with higher education has increased from 13.3% to 24% [22,23]. Moreover, after joining the EU in 2004, the inequality in income distribution has systematically decreased [24]. We can hypothesize that socio-economic changes taking place in Poland in the last years have made Poles’ eating behavior less dependent on education and financial situation, and more on the type of food available in popular chain stores, which are the main source of food purchases in Poland.

This study has practical implications for policymakers and public health authorities. First, this study underlines the urgent need to provide a nationwide campaign on risk factors of overweight/obesity and their consequences. Second, the sociodemographic factors associated with overweight/obesity that were identified in this study may allow personalizing educational campaigns on overweight/obesity. Third, characteristics of the nutritional status of adult Polish inhabitants presented in this study are a source of epidemiological data that may be used by policymakers and public health specialists to plan and develop health policy and long-term health strategies.

This study has several limitations. First, nutritional status was assessed based on the BMI. BMI does not capture body fat location information and cannot be used as an indicator of percent of body fat mass [54]. Nevertheless, the BMI is well-recognized and commonly used in population-based studies to assess weight status and classify overweight/obesity [11,12,31,32]. Secondly, classification to particular socio-economic groups is based on self-declared responses, so we can not exclude recall bias. Thirdly, in this study only, sociodemographic factors were assessed. Data on energy intake, alcohol consumption and smoking habits were not available during the preparation of this manuscript. Moreover, sociodemographic factors were defined based on self-reported data, so we cannot exclude the possibility of information bias. Nevertheless, data were collected by experienced interviewers to minimalize the risk of information bias. Nevertheless, this is the most up-to-date study on nutritional status based on the anthropometric measurements carried out in a large, representative sample of adults in Poland. This study was carried out between 2019 and 2020, and ended one month before the onset of the COVID-19 pandemic in Poland. Presented findings may be used in further comparative studies between different historical moments. Moreover, this is one of the first studies that provides detailed sociodemographic characteristics by nutritional status. The study was conducted according to the EFSA guidance on the EU Menu methodology [26,27], so the results of this study may be a good basis for international comparisons.

## 5. Conclusions

This study demonstrated a high prevalence of overweight and obesity among adults in Poland, wherein the prevalence of overweight and obesity is significantly higher among men. Out of 11 factors analyzed in this study, only gender, older age, occupational activity, living in rural areas, and having the chronic disease were significantly associated with greater odds of overweight/obesity. The lack of significant association between educational level and financial situation with overweight/obesity may indicate a diminishing role of sociodemographic factors in shaping the eating habits and nutritional status of the inhabitants of post-communist countries such as Poland, which have significantly developed over the last 30 years.

## Figures and Tables

**Table 1:** Characteristics of the sample by BMI status (*n* = 1811).

Variable	Total Sample *n* = 1811	BMI Status		
		Normal Weight *n* = 739	Overweight/Obese *n* = 1072	*p*
	*n* (%)	*n* (%)	*n* (%)	
**Gender**				
Male	911 (50.3)	282 (31.0)	629 (69.0)	<0.001
Female	900 (49.7)	457 (50.8)	443 (49.2)	
Age (years)				
18–29	372 (20.5)	246 (66.1)	126 (33.9)	<0.001
30–39	230 (12.7)	117 (50.9)	113 (49.1)	
40–49	188 (10.4)	69 (36.7)	119 (63.3)	
50–59	275 (15.2)	86 (31.3)	189 (68.7)	
60–69	312 (17.2)	89 (28.5)	223 (71.5)	
70+	434 (24.0)	132 (30.4)	302 (69.6)	
Marital status (*n* = 1803)				
single	478 (26.5)	271 (56.7)	207 (43.3)	<0.001
married	1012 (56.1)	356 (35.2)	656 (64.8)	
divorced	70 (3.9)	28 (40.0)	42 (60.0)	
widowed	243 (13.5)	83 (34.2)	160 (65.8)	
Place of residence (*n* = 1807)				
rural	742 (41.0)	289 (39.0)	453 (61.0)	0.19
urban	1065 (59.0)	448 (42.1)	617 (57.9)	
Educational level				
primary education	173 (9.6)	68 (39.3)	105 (60.7)	<0.001
vocational education	593 (32.7)	184 (31.0)	409 (69.0)	
secondary education	826 (45.6)	370 (44.8)	456 (55.2)	
higher education	219 (12.1)	117 (53.4)	102 (46.6)	
Occupational status				
active	938 (51.8)	417 (44.5)	521 (55.5)	0.001
passive	873 (48.2)	322 (36.9)	551 (63.1)	
Financial situation				
good or very good	739 (40.8)	351 (47.5)	388 (52.5)	<0.001
moderate	950 (52.5)	339 (35.7)	611 (64.3)	
bad or very bad	122 (6.7)	49 (40.2)	73 (59.8)	
Living conditions				
living alone	432 (23.9)	167 (38.7)	265 (61.3)	0.29
living with one or more persons	1379 (76.1)	572 (41.5)	807 (58.5)	
Having children under 18 years of age				
Yes	429 (23.7)	204 (47.6)	225 (52.4)	0.001
No	1382 (76.3)	535 (38.7)	847 (61.3)	
Presence of at least one chronic disease (*n* = 1800)				
yes	309 (17.2)	80 (25.9)	229 (74.1)	<0.001
no	1491 (82.8)	654 (43.9)	837 (56.1)	
Self-reported health status				
very good	344 (19.0)	209 (60.8)	135 (39.2)	<0.001
good	799 (44.1)	335 (41.9)	464 (58.1)	
moderate	596 (32.9)	175 (29.4)	421 (70.6)	
bad or very bad	72 (4.0)	20 (27.8)	52 (72.2)	
Physical activity level				
low	395 (21.8)	163 (41.3)	232 (58.7)	0.58
moderate	798 (44.1)	334 (41.9)	464 (58.1)	
high	618 (34.1)	242 (39.2)	376 (60.8)	

**Table 2:** The prevalence of overweight and obesity among males and females by sociodemographic factors.

	Male (*n* = 911)				Female (*n* = 900)			
Variable	Normal Weight *n* = 282	Overweight *n* = 478	Obesity *n* = 151	*p*	Normal Weight *n* = 457	Overweight *n* = 294	Obesity *n* = 149	*p*
	*n* (%)	*n* (%)	*n* (%)		*n* (%)	*n* (%)	*n* (%)	
Age (years)				<0.001				
18–29	97 (34.4)	16.1 (16.1)	14 (9.3)		149 (32.6)	27 (9.2)	8 (5.4)	<0.001
30–39	40 (14.2)	57 (11.9)	14 (9.3)		77 (16.8)	30 (10.2)	12 (8.0)	
40–49	27 (9.6)	51 (10.7)	19 (12.5)		42 (9.2)	39 (13.3)	10 (6.7)	
50–59	34 (12.1)	76 (15.9)	30 (19.9)		52 (11.4)	50 (17.0)	33 (22.2)	
60–69	30 (10.6)	94 (19.7)	32 (21.2)		59 (12.9)	53 (18.0)	44 (29.5)	
70+	54 (19.1)	123 (25.7)	42 (27.8)		78 (17.1)	95 (32.3)	42 (28.2)	
Marital status		*n* = 475	*n* = 150	<0.001	*n* = 456	*n* = 291		
single	119 (42.2)	105 (22.1)	30 (20.0)		152 (33.3)	50 (17.2)	22 (14.8)	<0.001
married	133 (47.2)	312 (65.7)	97 (64.7)		223 (48.9)	168 (57.7)	79 (53.0)	
divorced	7 (2.5)	13 (2.7)	6 (4.0)		21 (4.6)	17 (5.8)	6 (4.0)	
widowed	23 (8.2)	45 (9.5)	17 (11.3)		60 (13.2)	56 (19.3)	42 (28.2)	
Place of residence (*n* = 1807)	*n* = 280	*n* = 477	*n* = 150	0.30				
rural	111 (39.6)	199 (41.7)	71 (47.3)		178 (39.0)	127 (43.2)	56 (37.6)	0.40
urban	169 (60.4)	278 (58.3)	79 (52.7)		279 (61.0)	167 (56.8)	93 (62.4)	
Educational level				0.004				
primary education	35 (12.4)	38 (7.9)	14 (9.3)		33 (7.2)	35 (11.9)	18 (12.1)	<0.001
vocational education	85 (30.1)	197 (41.2)	73 (48.3)		99 (21.7)	90 (30.6)	49 (32.9)	
secondary education	135 (47.9)	203 (42.5)	57 (37.8)		235 (51.4)	133 (45.3)	63 (42.3)	
higher education	27 (9.6)	40 (8.4)	7 (4.6)		90 (19.7)	36 (12.2)	19 (12.7)	
Occupational status				0.76				
active	156 (55.3)	272 (56.9)	81 (53.6)		261 (57.1)	118 (40.1)	50 (33.6)	<0.001
passive	126 (44.7)	206 (43.1)	70 (46.4)		196 (42.9)	176 (59.9)	99 (66.4)	
Financial situation				0.02				
good or very good	138 (49.0)	178 (37.3)	54 (35.8)		213 (46.6)	107 (36.4)	49 (32.9)	0.01
moderate	129 (45.7)	275 (57.5)	88 (58.3)		210 (46.0)	162 (55.1)	86 (57.7)	
bad or very bad	15 (5.3)	25 (5.2)	9 (5.9)		34 (7.4)	25 (8.5)	14 (9.4)	
Living conditions				0.04				
living alone	61 (21.6)	83 (17.4)	40 (26.5)		106 (23.2)	85 (28.9)	57 (38.3)	0.001
living with one or more persons	221 (78.4)	395 (82.6)	111 (73.5)		351 (76.8)	209 (71.1)	92 (61.7)	
Having children under 18 years of age				0.09				
Yes	67 (23.8)	108 (22.6)	23 (15.2)		137 (30.0)	73 (24.8)	21 (14.1)	<0.001
No	215 (76.2)	370 (77.4)	128 (84.8)		320 (70.0)	221 (75.2)	128 (85.9)	
Presence of at least one chronic disease (*n* = 1800)	*n* = 281	*n* = 476		<0.001	*n =* 453	*n* = 291	*n* = 148	
yes	26 (9.3)	80 (16.8)	38 (25.2)		54 (11.9)	70 (24.1)	41 (27.7)	<0.001
no	255 (90.8)	396 (83.2)	113 (74.8)		399 (88.1)	221 (75.9)	107 (72.3)	
Self-reported health status				<0.001				
very good	77 (27.3)	81 (17.0)	12 (8.0)		132 (28.9)	31 (10.5)	11 (7.4)	<0.001
good	127 (45.0)	215 (45.0)	62 (41.0)		208 (45.5)	130 (44.2)	57 (38.2)	
moderate	65 (23.1)	165 (34.5)	69 (45.7)		110 (24.1)	117 (39.8)	70 (47.0)	
bad or very bad	13 (4.6)	17 (3.5)	8 (5.3)		7 (1.5)	16 (5.4)	11 (7.4)	
Physical activity level				0.14				
low	65 (23.0)	98 (20.5)	20 (13.3)		98 (21.5)	81 (27.6)	33 (22.1)	0.07
moderate	115 (40.8)	217 (45.4)	73 (48.3)		219 (47.9)	110 (37.4)	64 (43.0)	
high	102 (36.2)	163 (34.1)	58 (38.4)		140 (30.6)	103 (35.0)	52 (34.9)	

**Table 3:** Physical activity level by BMI status and gender (*n* = 1811).

	BMI Status: Normal Weight			BMI Status: Overweight/Obese		
Variable	Male *n* = 282	Female *n* = 457	*p*	Male *n* = 629	Female *n* = 443	*p*
	*n* (%)	*n* (%)		*n* (%)	*n* (%)	
Physical activity level			0.08			
low	65 (23.0)	98 (21.5)		118 (18.8)	114 (25.7)	0.01
moderate	115 (40.8)	219 (47.9)		290 (46.1)	174 (39.3)	
high	102 (36.2)	140 (30.6)		221 (35.1)	155 (35.0)	

**Table 4:** Odds ratios (OR) and 95% confidence intervals (CI) for overweight and obesity compared with normal weight to selected sociodemographic factors in a representative sample of adults in Poland.

Variable	Univariate Logistic Regression		Unrestricted Multivariate Logistic Regression		Restricted Multivariate Logistic Regression	
	% (95% CI)	*p*	% (95% CI)	*p*	% (95% CI)	*p*
Age (years)						
	1.03 (1.02–1.03)	<0.001	1.03 (1.02–1.04)	<0.001	1.04 (1.03–1.04)	<0.001
Gender						
Male	2.30 (1.90–2.79)	<0.001	2.44 (1.96–3.00)	<0.001	2.44 (2.00–2.99)	<0.001
Female	Reference		Reference		Reference	
Marital status						
Ever married	2.41 (1.94–2.98)	<0.001	1.03 (0.96–1.77)	0.09		
Never narried	Reference		Reference			
Having children under 18 years of age						
Yes	Reference		Reference			
No	1.44 (1.15–1.79)	0.001	0.92 (0.70–1.21)	0.56		
Educational level						
primary education	1.77 (1.18–2.65)	0.006	0.89 (0.54–1.47)	0.65		
vocational education	2.55 (1.86–3.50)	<0.001	1.22 (0.85–1.76)	0.29		
secondary education	1.41 (1.05–1.91)	0.02	1.04 (0.75–1.44)	0.80		
higher education	Reference		Reference			
Occupational status						
active	0.73 (0.61–0.88)	0.001	1.36 (1.04–1.77)	0.02	1.50 (1.17–1.93)	0.001
passive	Reference		Reference		Reference	
Financial situation						
good or very good	Reference		Reference			
moderate	1.63 (1.34–1.98)	<0.001	1.21 (0.97–1.51)	0.09		
bad or very bad	1.35 (0.91–1.99)	0.13	0.90 (0.56–1.44)	0.66		
Place of residence						
rural	1.14 (0.94–1.38)	0.19	1.29 (1.04–1.60)	0.02	1.32 (1.07–1.63)	0.008
urban	Reference		Reference		Reference	
Living conditions						
living alone	1.13 (0.90–1.40)	0.30	0.92 (0.70–1.22)	0.62		
living with one or more persons	Reference		Reference			
Presence of at least one chronic disease						
yes	2.24 (1.70–2.94)	<0.001	1.54 (1.19–2.21)	0.008	1.51 (1.11–2.07)	0.009
no	Reference		Reference		Reference	
Physical activity level						
low	0.92 (0.71–1.19)	0.50	0.93 (0.71–1.23)	0.62		
moderate	0.89 (0.72–1.11)	0.31	0.89 (0.70–1.12)	0.31		
high	Reference		Reference			

## Data Availability

Data are available on reasonable request. The dataset used to conduct the analyses is available from corresponding author on reasonable request.
